# Standardised spider (Arachnida, Araneae) inventory of Kilpisjärvi, Finland

**DOI:** 10.3897/BDJ.8.e56486

**Published:** 2020-09-17

**Authors:** Niina Kiljunen, Timo Pajunen, Caroline Fukushima, Arttu Soukainen, Jaakko Kuurne, Tuuli Korhonen, Joni Saarinen, Ilari Falck, Erkka Laine, Stefano Mammola, Fernando Urbano, Nuria Macías-Hernández, Pedro Cardoso

**Affiliations:** 1 Department of Biological and Environmental Science, University of Jyväskylä, Jyväskylä, Finland Department of Biological and Environmental Science, University of Jyväskylä Jyväskylä Finland; 2 Laboratory for Integrative Biodiversity Research (LIBRe), Finnish Museum of Natural History (LUOMUS), University of Helsinki, Helsinki, Finland Laboratory for Integrative Biodiversity Research (LIBRe), Finnish Museum of Natural History (LUOMUS), University of Helsinki Helsinki Finland; 3 Finnish Museum of Natural History (LUOMUS), University of Helsinki, Helsinki, Finland Finnish Museum of Natural History (LUOMUS), University of Helsinki Helsinki Finland; 4 Molecular Ecology Group (MEG), Water Research Institute, National Research Council (CNR-IRSA), Verbania Pallanza, Italy Molecular Ecology Group (MEG), Water Research Institute, National Research Council (CNR-IRSA) Verbania Pallanza Italy

**Keywords:** Arthropoda, biogeography, Lapland, subarctic

## Abstract

**Background:**

A spider taxonomy and ecology field course was organised in Kilpisjärvi Biological Station, northern Finland, in July 2019. During the course, four 50 × 50 m plots in mountain birch forest habitat were sampled following a standardised protocol. In addition to teaching and learning about spider identification, behaviour, ecology and sampling, the main aim of the course was to collect comparable data from the Kilpisjärvi area as part of a global project, with the purpose of uncovering global spider diversity patterns.

**New information:**

A total of 2613 spiders were collected, of which 892 (34%) were adults. Due to uncertainty of juvenile identification, only adults are included in the data presented in this paper. The observed adult spiders belong to 51 species, 40 genera and 11 families, of which the Linyphiidae were the most rich and abundant with 28 (55%) species and 461 (52%) individuals. Lycosidae had six species and 286 individuals, Gnaphosidae five species and 19 individuals, Thomisidae four species and 24 individuals, Theridiidae two species and 23 individuals. All other six families had one species and less than 40 individuals. The most abundant species were the linyphiid *Agnyphantes expunctus* (204) and the lycosids *Pardosa eiseni* (164) and *Pardosa hyperborea* (107).

## Introduction

The Kilpisjärvi area in Lapland belongs to the hemi-arctic climate zone and north boreal vegetation zone (Kontula and Raunio 2018). The tundra habitat with no arboreal vegetation dominates in the region, but scattered mountain birch (Betula pubescens subsp. czerepanovii, N. I. Orlova, 1987) forests are abundant (Pääkkö et al. 2018). According to the International Union for the Conservation of Nature Red List of Ecosystems Criteria, mountain-birch forests are categorised as vulnerable (VU) in Finland (Pääkkö et al. 2018). The forest understorey commonly consists of species, such as *Betula nana* (L., 1753) *Empetrum nigrum* (L., 1753), *Vaccinium myrtillus* (L., 1753), *Vaccinium vitis-idaea* (L., 1753) *Cornus suecica* (L., 1753), mosses and lichens. During the last glacial maximum, the whole area of Finland was covered with glacial ice. The ice cover retreated from northernmost Lapland about 10,000 years ago, when the ongoing process of species colonisation of the area started.

The Finnish biota is well known due to its strong tradition in taxonomic work (Hyvärinen et al. 2019, Rassi et al. 2010, Rassi et al. 2000, Rassi et al. 1992, Rassi et al. 1986, Koponen 2010). Currently, approximately 45,000 species are known to occur in Finland, but the real number is estimated to be about 48,000 according to the latest Finnish assessment of threatened species (Hyvärinen et al. 2019). In addition, the Finnish fauna has been extensively assessed twice according to the IUCN criteria (Rassi et al. 2000, Rassi et al. 2010). Such assessments were only possible due to long-term monitoring of species (Rassi et al. 1986, Rassi et al. 1992). The Finnish Expert Group on Araneae (https://finaraneae.org/) accomplished the assessment of threatened spiders in 2019 (Pajunen et al. 2019). This group is also maintaining the Checklist of Finnish Spiders, that includes 647 species (http://biolcoll.utu.fi/arach/checklist_of_spiders_in_Finland.htm) (Koponen et al. 2016). Seppo Koponen recently described the history of arachnology in Finland (Koponen 2010).

**Figure 1.**
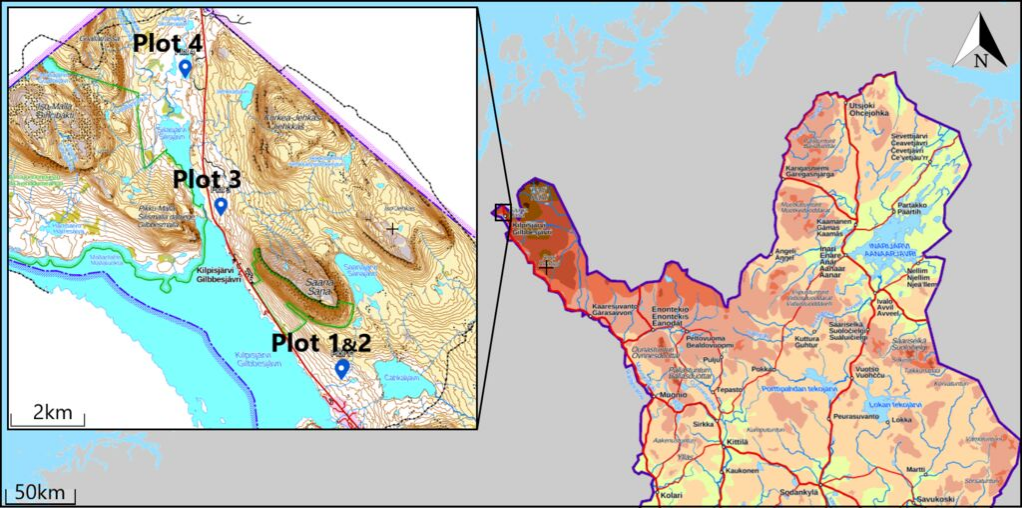
Location of the four sampled plots in northern Finland (data from https://asiointi.maanmittauslaitos.fi/karttapaikka/).

During the "Advanced course in identification of species: Spider taxonomy and ecology" of the Ecology & Evolutionary Biology Masters' programme at the Faculty of Biological and Environmental Sciences, University of Helsinki, the authors were teaching and learning about spider identification, taxonomy, ecology and sampling. We sampled four plots in mountain birch forest in Kilpisjärvi (Fig. 1). The plots sampled in this area are so far the northernmost and only plots sampled from the hemi-arctic zone in a global spider diversity project (see http://biodiversityresearch.org/research/biogeography/). To produce high-quality data comparable with other sampling conducted under the global project, sampling was performed following the standardised COBRA – Conservation Oriented Biodiversity Rapid Assesment – protocol (Cardoso 2009). By following the protocol, it is ensured that these data can be re-used in multiple ways.

## Sampling methods

### Study extent

**Figure 2.**
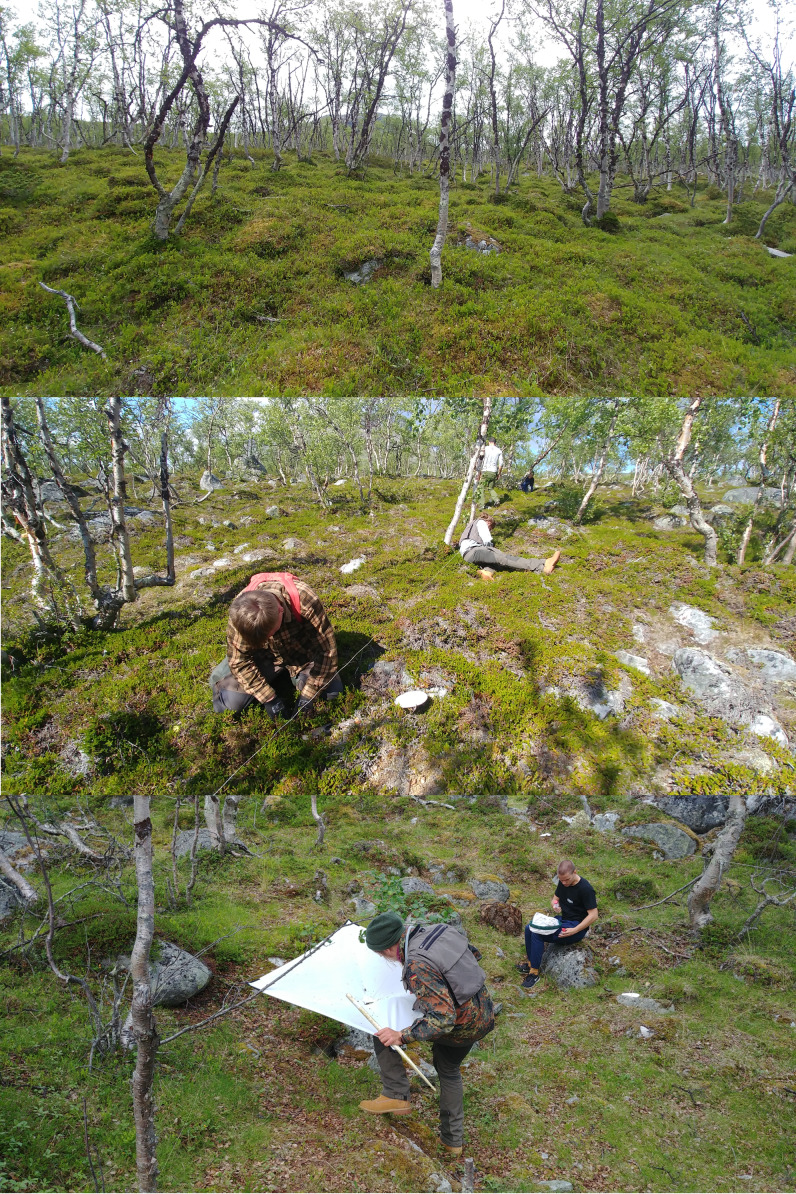
Views of plot 3 (top), pitfall trap sampling at plot 1 (middle) and active sampling at plot 4 (bottom) (photos by Pedro Cardoso).

Four 50 × 50 m mountain birch forest plots were chosen for sampling (Table 1;Fig. 2). These were chosen as on other occasions (Malumbres-Olarte et al. 2016, Cardoso et al. 2017) roughly following a log scale in interplot distances. Plots 1 and 2 were located in dry *Empetrum*-*Myrtillus* mountain birch forest with rocky base, approximately 100 m apart from each other. Plot 3 was located in mesic *Cornus*-*Myrtillus* mountain birch forest, circa 3 km from the previous plots and plot 4 was located in low-herb mountain birch forest, circa 6 km from the first. Interplot distances were also dictated by availability of similar habitats in similar altitudes to avoid confounding factors in future analyses.

### Sampling description

Sampling at each of the four studied plots was performed following the COBRA protocol. This protocol includes 12 h of active sampling and 12 h of pitfall effort with 48 pitfall traps in each study plot (pitfall traps are distributed in 12 samples). Active sampling includes night aerial sampling (4 h/plot), day/night sweeping (2 h/plot each) and day/night beating (2 h/plot each) (Fig. 2). This protocol was first proposed and described in detail by Cardoso (2009) and recently applied and adapted to the tropics (Malumbres-Olarte et al. 2016) and islands (Emerson et al. 2016). This study follows two similar inventories previously performed in Finland, specifically Hankoniemi (in 2016, coordinates 59.8 and 59.9 Latitude; 23.0 and 23.3 Longitude) (Cardoso et al. 2017) and Lammi (in 2019, coordinates 61.05 and 61.06 Latitude; 25.04 and 25.05 Longitude) (Soukainen et al. 2020).

**Study dates**: Sampling was carried out in July and August 2019. Pitfall traps were set on 22 July and collected on 5 August. Active sampling was performed between 22 and 24 July.

## Geographic coverage

### Description

Kilpisjärvi, Finland

### Coordinates

69.02 and 69.09 Latitude; 20.74 and 20.87 Longitude.

## Taxonomic coverage

### Taxa included

**Table:** 

Rank	Scientific Name	Common Name
order	Araneae	Spiders

## Temporal coverage

**Data range:** 2019-7-22 – 2019-8-05.

## Usage rights

### Use license

Open Data Commons Attribution License

## Data resources

### Data package title

COBRA_Finland_Kilpisjärvi

### Resource link

http://ipt.pensoft.net/resource?r=cobra_finland_kilpisjarvi

### Alternative identifiers

https://doi.org/10.15468/425g9e

### Number of data sets

1

### Data set 1.

#### Data set name

COBRA_Finland_Kilpisjärvi

#### Number of columns

27

#### Description

These same data are also available through the Finnish Biodiversity Information Facility (http://www.laji.fi) at the following links:

http://tun.fi/JX.1136721

http://tun.fi/JX.1136722

http://tun.fi/JX.1136723

http://tun.fi/JX.1136724

http://tun.fi/JX.1136725

http://tun.fi/JX.1136726

http://tun.fi/JX.1136727

http://tun.fi/JX.1136728

http://tun.fi/JX.1136729

http://tun.fi/JX.1136730

**Data set 1.** 

Column label	Column description
occurrenceID	An identifier for the Occurrence (as opposed to a particular digital record of the occurrence).
basisOfRecord	The specific nature of the data record.
recordedBy	A list (concatenated and separated) of names of people, groups or organisations responsible for recording the original Occurrence.
individualCount	The number of individuals represented present at the time of the Occurrence.
lifeStage	The age class or life stage of the biological individual(s) at the time the Occurrence was recorded.
samplingProtocol	The name of, reference to, or description of the method or protocol used during an event.
eventRemarks	Comments or notes about the Event.
locationID	An identifier for the set of location information (data associated with dcterms:Location).
country	The name of the country or major administrative unit in which the location occurs.
county	The full, unabbreviated name of the next smaller administrative region than stateProvince (county, shire, department etc.) in which the location occurs.
locality	The specific description of the place.
minimumElevationInMetres	The lower limit of the range of elevation (altitude, usually above sea level), in metres.
maximumElevationInMetres	The upper limit of the range of elevation (altitude, usually above sea level), in metres.
decimalLatitude	The geographic latitude (in decimal degrees, using the spatial reference system given in geodeticDatum) of the geographic centre of a location.
decimalLongitude	The geographic longitude (in decimal degrees, using the spatial reference system given in geodeticDatum) of the geographic centre of a location.
geodeticDatum	The ellipsoid, geodetic datum or spatial reference system (SRS) upon which the geographic coordinates given in decimalLatitude and decimalLongitude as based.
coordinateUncertaintyInMetres	The horizontal distance (in metres) from the given decimalLatitude and decimalLongitude describing the smallest circle containing the whole of the Location.
identifiedBy	A list (concatenated and separated) of names of people, groups or organisations who assigned the taxon to the subject.
dateIdentified	The date on which the subject was identified as representing the taxon.
kingdom	The full scientific name of the kingdom in which the taxon is classified.
phylum	The full scientific name of the phylum or division in which the taxon is classified.
class	The full scientific name of the class in which the taxon is classified.
order	The full scientific name of the order in which the taxon is classified.
family	The full scientific name of the family in which the taxon is classified.
genus	The full scientific name of the genus in which the taxon is classified.
scientificName	The full scientific name, with authorship and date information, if known.
taxonRank	The taxonomic rank of the most specific name in the scientificName.

## Additional information

A total of 2613 spiders were sampled, of which 892 (34%) were adults. Due to uncertainty of juvenile identification, only adults are discussed in this paper and included in the dataset (see Cardoso 2020). Observed adult spiders belong to 51 species, 40 genera and 11 families (Table 2 and specimens are deposited at the Finnish Museum of Natural History Luomus). Of these, 28 species (55%) were Linyphiidae, six (12%) Lycosidae, five (10%) Gnaphosidae, four (8%) Thomisidae and two (4%) Theridiidae. All the other six families had only one species represented. Linyphiidae was the most abuntant family with 461 adult individuals captured (52%), followed by Lycosidae (286; 32%) and Cybaeidae (38; 4%). All the other families had less than 30 individuals. The most abundant species amongst adult spiders were the linyphiid *Agnyphantes expunctus* with 204 individuals (23% of all adult individals in the inventory) and the lycosids *Pardosa eiseni* (164; 18%) and *Pardosa hyperborea* (107; 12%). All other species had less than 60 individuals. Plot 3 had the highest species richness (33), with 65% of all the species collected, both plot 1 and plot 2 had 28 species (55%) and plot 4 had 27 species (53%).

**Discussion**

The majority of species collected from Kilpisjärvi area are common and widespread either in the whole of Finland or in the northernmost parts of the country. The only exception is the gnaphosid *Micaria tripunctata*, recorded for the second time in Finland (Holm 1978). *Micaria tripunctata* can be considered as a northern species in Finland, as it has been found only in this region (Koponen et al. 2013). *Agnyphantes expunctus* was the most abundant species of the inventory. This species has been previously found from pine (Nekhaeva 2016), spruce (Palmgren 1977) and birch (Nekhaeva 2015) forests and also from open, semi-open and semi-moistured areas (Matveinen-Huju 2004). This has also been considered as a northern species in Finland since 1977 (Palmgren 1977). Kilpisjärvi belongs to its known range and has plenty of suitable habitats for the species, which can explain the large abundance. *Pardosa eiseni* and *P. hyperborea* were also notably abundant in this inventory. *Pardosa hyperborea* has been previously observed to be abundant in birch forest and tundra (Nekhaeva 2016). Both *P. eiseni* and *P. hyperborea* are noticed to be abundant also in the Kevo area (Koponen 1975). *Pardosa eiseni* is considered as a northern species according to the Atlas of Araneae of Finland (Koponen et al. 2013). In addition to *P. eiseni* and *M. tripunctata*, at least five other species sampled can be considered as northern species in Finland (Koponen et al. 2013): *Baryphyma trifrons*, *Decipiphantes decipiens*, *Gnaphosa sticta*, *Macrargus multesimus* and *Micaria alpina*.

Of the collected adult individuals, 318 were caught during nocturnal sampling and 166 during diurnal sampling. The remaining 408 individuals, belonging to 36 species, were caught with pitfall traps. The species observed with pitfall traps clearly differ from the species caught by using active methods. The majority of the individuals caught with pitfall traps were lycosids and only very few lycosid individuals were caught with active methods. With nocturnal active sampling, 21 species were observed, whereas with diurnal active sampling, the number of observed species was 16. We must note, however, that aerial sampling was done only during the night and these numbers cannot be easily compared. If we compare only the results from sweeping and beating, the number of observed individuals and species differ only very slightly between night and day. With nocturnal sweeping, the number of caught individuals is 64, belonging to 12 species and with beating 151 (11), whereas with diurnal sweeping, the number is 62 (10 species) and diurnal beating 102 (11). Species composition between day and night were also very similar. Five species, *Cryphoeca silvicola*, *Hypomma bituberculatum*, *Nuctenea silvicultrix*, *Thyreosthenius parasiticus* and *Xysticus cristatus* were observed only at night. *Ceratinella wideri* and *Tenuiphanthes alacris* were observed only during daytime. The remaining 14 species were observed both during day and night, but often the number of observed individuals was higher at night. These numbers indicate that adult spiders might be slightly more active at night-time, despite the light level being only slightly different from daytime during the polar day. The differences are, however, small and this pattern might be spurious.

Species diversity obtained in this inventory (51 species) considerably differs from the two recently-performed inventories in Finland, where the same standardised COBRA protocol was used. In Hankoniemi, southernmost Finland, 104 species were captured in four forest plots (Cardoso et al. 2017) and in Lammi, southern Finland, 115 species were found in three plots, two forest and one grassland plot (Soukainen et al. 2020). The number of observed families was also higher in the south. The majority of the species (over 65%) observed in Kilpisjärvi were not observed in the southern sites, including the ones that are considered as northern species. The majority of the species observed in both Kilpisjärvi and the southern sites belong to the family Linyphiidae; however, more than half of the Linyphiidae species observed in Kilpisjärvi were not observed in the south. Hahniidae was the only family found from Kilpisjärvi. In contrast, Clubionidae and Tetragnathidae were the most species-rich of the families found only from southern sites. In Kilpisjärvi and Hankoniemi (Cardoso et al. 2017), lycosids were abundant in all four plots considered as forest habitats, whereas in Lammi, the vast majority of the lycosids were caught on the grassland plot and almost none in the two forest plots (Soukainen et al. 2020). The number of compared plots is very limited; however, the difference is clear and can be due to very different forest habitats between Lammi and the two other sites. The two inventories in southern Finland were performed in June, whereas this inventory in Kilpisjärvi was performed at the end of July. Difference in timing might partially affect the differences in the observed species compositions between these three sites. In addition, Kilpisjärvi is located over 900 km further north than Lammi and over 1000 km further north than Hankoniemi. The difference in numbers of observed species and species compositions can be due to a very strong latitudinal diversity gradient (Hillebrand 2004). Additionally, the altitude of sampled plots in Kilpisjärvi is 400 to 500 m higher than the southern sites, further contributing to more extreme environmental conditions and decreasing the number of observed species (MacArthur 1972, Rahbek 1995).

## Figures and Tables

**Table 1.** Coordinates of sampling plots (WGS84).

Plot	Habitat	decimalLatitude, decimalLongitude	Metres above sea level
1	dry *Empetrum*-*Myrtillus* mountain birch forest	69.023448, 20.872026	520-530
2	dry *Empetrum*-*Myrtillus* mountain birch forest	69.024366, 20.872175	520-530
3	mesic *Cornus*-*Myrtillus* mountain birch forest	69.060888, 20.777347	510-520
4	low-herb mountain birch forest	69.093133, 20.744404	510-520

**Table 2.** Richness and abundance of species per plot (adults only).

Family	Species	Plot 1	Plot 2	Plot 3	Plot 4	Total
Araneidae	*Nuctenea silvicultrix* (C. L. Koch, 1835)		4	1		5
Cybaeidae	*Cryphoeca silvicola* (C. L. Koch, 1834)	20	10	8		38
Gnaphosidae	*Gnaphosa lapponum* (L. Koch, 1866)	2	2		3	7
Gnaphosidae	*Gnaphosa microps* (Holm, 1939)		1	2	4	7
Gnaphosidae	*Gnaphosa sticta* (Kulczynski, 1908)		3			3
Gnaphosidae	*Micaria alpina* (L. Koch, 1872)	1				1
Gnaphosidae	*Micaria tripunctata* (Holm, 1978)			1		1
Hahniidae	*Hahnia ononidum* (Simon, 1875)		2	5		7
Linyphiidae	*Agnyphantes expunctus* (O. P.-Cambridge, 1875)	20	16	61	107	204
Linyphiidae	*Agyneta cauta* (O. P.-Cambridge, 1902)			1		1
Linyphiidae	*Baryphyma trifrons* (O. P.-Cambridge, 1863)				7	7
Linyphiidae	*Bolephthyphantes index* (Thorell, 1856)	1	2	1	31	35
Linyphiidae	*Bolyphantes luteolus* (Blackwall, 1833)	5	3	43	4	55
Linyphiidae	*Ceratinella wideri* (Thorell, 1871)				1	1
Linyphiidae	*Decipiphantes decipiens* (L. Koch, 1879)	1				1
Linyphiidae	*Diplocentria bidentata* (Emerton, 1882)			5		5
Linyphiidae	*Entelecara erythropus* (Westring, 1851)	1	8	1	5	15
Linyphiidae	*Hilaira herniosa* (Thorell, 1875)	3	3	2	1	9
Linyphiidae	*Hypomma bituberculatum* (Wider, 1834)			10		10
Linyphiidae	*Macrargus multesimus* (O. P.-Cambridge, 1875)				1	1
Linyphiidae	*Macrargus rufus* (Wider, 1834)	1				1
Linyphiidae	*Maso sundevalli* (Westring, 1851)	7	1		1	9
Linyphiidae	*Micrargus herbigradus* (Blackwall, 1854)		1			1
Linyphiidae	*Obscuriphantes obscurus* (Blackwall, 1841)	2		12	17	31
Linyphiidae	*Oedothorax* sp.				1	1
Linyphiidae	*Oreonetides vaginatus* (Thorell, 1872)			1	1	2
Linyphiidae	*Oryphantes angulatus* (O. P.-Cambridge, 1881)			1		1
Linyphiidae	*Palliduphantes antroniensis* (Schenkel, 1933)	1				1
Linyphiidae	*Pelecopsis mengei* (Simon, 1884)	2	2	3	4	11
Linyphiidae	*Porrhomma pallidum* (Jackson, 1913)	1		2		3
Linyphiidae	*Tenuiphantes alacris* (Blackwall, 1853)				1	1
Linyphiidae	*Tenuiphantes mengei* (Kulczynski, 1887)	2	2	10	4	18
Linyphiidae	*Tenuiphantes tenebricola* (Wider, 1834)			2	2	4
Linyphiidae	*Thyreosthenius parasiticus* (Westring, 1851)	2		2		4
Linyphiidae	*Walckenaeria cuspidata* (Blackwall, 1833)			2		2
Linyphiidae	*Zornella cultrigera* (L. Koch, 1879)	12	3	4	8	27
Lycosidae	*Alopecosa aculeata* (Clerck, 1757)		1	2	1	4
Lycosidae	*Alopecosa taeniata* (C. L. Koch, 1835)	1				1
Lycosidae	*Pardosa amentata* (Clerck, 1757)			4		4
Lycosidae	*Pardosa eiseni* (Thorell, 1875)	45	54	43	22	164
Lycosidae	*Pardosa hyperborea* (Thorell, 1872)	33	19	38	17	107
Lycosidae	*Pardosa lugubris* (Walckenaer, 1802)	2		4		6
Miturgidae	*Zora nemoralis* (Blackwall, 1861)	1	1		1	3
Philodromidae	*Thanatus formicinus* (Clerck, 1757)		1			1
Salticidae	*Evarcha falcata* (Clerck, 1757)	4	16	4	1	25
Theridiidae	*Ohlertidion ohlerti* (Thorell, 1870)	1	4	1		6
Theridiidae	*Robertus scoticus* (Jackson, 1914)	5	1	10	1	17
Thomisidae	*Ozyptila atomaria* (Panzer, 1801)	2	3			5
Thomisidae	*Xysticus audax* (Schrank, 1803)		4	2	1	7
Thomisidae	*Xysticus cristatus* (Clerck, 1757)		1	3		4
Thomisidae	*Xysticus obscurus* (Collett, 1877)	5	1		2	8
	**Species richness**	**28**	**28**	**33**	**27**	**51**
	**Individuals**	**183**	**169**	**291**	**249**	**892**
